# Associations of Bone Turnover Markers with Cognitive Function in Patients Undergoing Hemodialysis

**DOI:** 10.1155/2020/8641749

**Published:** 2020-04-24

**Authors:** Ping-Hsun Wu, Yi-Ting Lin, Cheng-Sheng Chen, Yi-Wen Chiu, Jer-Chia Tsai, Po-Lin Kuo, Ya-Ling Hsu, Östen Ljunggren, Bengt Fellström, Mei-Chuan Kuo

**Affiliations:** ^1^Graduate Institute of Clinical Medicine, College of Medicines, Kaohsiung Medical University, Kaohsiung, Taiwan; ^2^Faculty of Medicine, College of Medicine, Kaohsiung Medical University, Kaohsiung, Taiwan; ^3^Division of Nephrology, Department of Internal Medicine, Kaohsiung Medical University Hospital, Kaohsiung, Taiwan; ^4^Department of Medical Sciences, Uppsala University, Uppsala, Sweden; ^5^Department of Family Medicine, Kaohsiung Medical University Hospital, Kaohsiung, Taiwan; ^6^Department of Psychiatry, Kaohsiung Medical University Hospital, Kaohsiung Medical University, Kaohsiung, Taiwan; ^7^Faculty of Renal Care, College of Medicine, Kaohsiung Medical University, Kaohsiung, Taiwan; ^8^Graduate Institute of Medicine, College of Medicine, Kaohsiung Medical University, Kaohsiung, Taiwan

## Abstract

**Background:**

Patients undergoing hemodialysis experience a greater risk of cognitive impairment than the general population, but limited data elucidates the biomarkers on this. We evaluated the association of bone turnover markers on cognitive function among 251 prevalent hemodialysis enrollees in a cross-sectional study.

**Methods:**

251 hemodialysis patients (median age = 57.8, 55% men) and 37 control subjects (mean age = 61.2, 56% men) without a prior stroke or dementia diagnosis were enrolled. Serum concentrations of 8 bone markers were analyzed as the association of cognitive function (Montreal Cognitive Assessment (MoCA) and Cognitive Abilities Screening Instrument (CASI)) using linear regression analysis.

**Results:**

A lower cognitive function was noted in hemodialysis patients compared to control subjects. The receptor activator of nuclear factor kappa-B ligand (RANKL) was the only bone marker found to be associated with cognitive function (MoCA and CASI tests) in hemodialysis patients without a prior stroke or dementia diagnosis. In stepwise multiple linear regression analysis, the association remained significant in MoCA (*β* = 1.14, 95% CI 0.17 to 2.11) and CASI (*β* = 3.06, 95% CI 0.24 to 5.88). Short-term memory (*β* = 0.52, 95% CI 0.01 to 1.02), mental manipulation (*β* = 0.51, 95% CI 0.05 to 0.96), and abstract thinking (*β* = 0.57, 95% CI 0.06 to 1.09) were the significant subdomains in the CASI score related to RANKL.

**Conclusions:**

Serum RANKL levels were potentially associated with better cognitive function in hemodialysis patients. Further large-scale and prospective studies are needed to confirm our findings.

## 1. Introduction

Patients with end-stage kidney disease (ESKD) have a threefold dementia prevalence rate than the age-matched general population [1]. ESKD patients with dementia comorbidity further worsen the adverse outcomes, such as hospitalization, mortality, and dialysis withdrawal [2, 3]. Cerebrovascular disease, anemia, secondary hyperparathyroidism, dialysis disequilibrium, and uremic toxins were the major causes of cognitive impairment in ESKD patients [4, 5]. Although ESKD patients share the same risk factors for dementia as the general population, these are not sufficient to completely explain the cognitive impairment and dementia on them; hence, the possibility of identifying novel mechanisms and disease biomarkers is suggested.

Osteoporosis and low bone mineral density (osteopenia) have been associated with cognitive impairment and dementia [6–9]. This raises the possibility that factors related to bone regulation and function may influence cognitive performance. Because bone-related peptides are secreted into the circulation, there has been growing interest in their influences on the central nervous system [10]. Several bone turnover markers were found to be associated with cognitive function in general population, such as Dickkopf-related protein 1 (DKK1) [11, 12], Osteocalcin (OC) [13, 14], Osteopontin (OPN) [15], Osteoprotegerin (OPG) [16], and Leptin [17]. These findings suggested that there might be a convergence in mechanisms between bone and neurodegeneration and that bone may possess endocrine properties which aid in maintaining cognitive well-being.

The discovery of novel risk markers for cognitive impairment on ESKD patients can help us to predict the trait and has the potential to provide us with a better understanding of the pathogenesis. Recent technological advances have enabled the simultaneous measurement of multiple proteins to provide new opportunities for unbiased discovery of novel pathophysiologic pathways of disease, as well as for the identification of novel clinically relevant biomarkers. We aimed to explore the potential biomarkers of cognitive function by using a Luminex bead-based multiplex assay on ESKD patients. In the present study, we used these data from a study of 251 prevalent hemodialysis patients to compare the association between cognitive function and bone turnover biomarkers.

## 2. Materials and Methods

### 2.1. Subjects

From August 2016 to January 2017, we recruited participants from 2 hemodialysis units (Kaohsiung Municipal Hsiao-Kang Hospital and Kaohsiung Medical University Hospital) in Taiwan. Eligible participants were those who had an age > 30 years and who were receiving hemodialysis for at least 90 days. Participants with cerebrovascular disease (*N* = 39) or dementia (*N* = 6) were excluded. A total of 293 individuals were screened, and neuropsychological tests were performed on them. Those subjects who failed to complete the neuropsychological tests were excluded from the final analysis (*N* = 45) (Supplementary Figure 1). All participants received regular hemodialysis three times per week through the use of automated volumetric machines. Each hemodialysis session lasted 3.5-4 hours and involved using high-flux dialyzers. The blood flow rate was controlled between 250 and 300 ml/min, and the dialysate flow was maintained at 500 ml/min, and single pool *Kt*/*V*was more than 1.2 per week. Informed consent was obtained from all subjects. The study protocol was approved by the Institutional Review Board of Kaohsiung Medical University (KMUHIRB-E(I)-20160095).

### 2.2. Cognitive Function Assessment

Cognitive function tests were assessed in the study: Cognitive Abilities Screening Instrument (CASI) [18–20] and Montreal Cognitive Assessment (MoCA) [21] (Supplementary Table 1). The CASI is a 40-item global cognitive test that assesses a broad range of cognitive domains. Nine cognitive evaluation domains were evaluated including attention, orientation, language, list-generating fluency, mental manipulation, abstraction/judgment, drawing, long-term memory, and short-term memory. The MoCA is a 30-point screening tool and encompasses multiple cognitive domains such as language, attention, visuospatial abilities and executive function, concentration and working memory, short-term memory, and orientation to time and place. The MoCA has been translated and adapted into several languages and is available freely on the Internet (http://www.mocatest.org). The tasks include cube copying, clock drawing, naming, digit span backward and forward, serial subtraction, selective attention, sentence repetition, phonemic word fluency, verbal abstraction, a 5-word learning and delayed recall task, and spatial and temporal orientation. Besides, we also performed the Center for Epidemiologic Studies Depression Scale (CES-D) to determine depression symptoms' score, as part of a broader neurocognitive assessment.

### 2.3. Multiplex Analysis of Serum Biomarkers

Serum was obtained using a serum separation tube (SST) after blood centrifugation at 1500 rpm for 10 min at 4°C. Samples were aliquoted and stored at -80°C until use. The MILLIPLEX MAP technology (Luminex) combines the principle of a sandwich immunoassay with the fluorescent bead-based technology allowing individual and multiplex analyses of different analytes in a single microtiter well. Defrosted serum samples were analyzed using a multiplex immunobead assay (Millipore, St. Charles, MO, US) to simultaneously quantify multiple potential biomarkers. Human RANKL Single Plex (receptor activator of nuclear factor kappa-Β ligand (RANKL)) and MILLIPLEX MAP Human Bone Panel (Dickkopf-related protein 1 (DKK1), Fibroblast growth factor 23 (FGF23), Leptin, Osteocalcin (OC), Osteopontin (OPN), Osteoprotegerin (OPG), and Sclerostin (SOST)) were used for determining the serum levels of these molecules (Supplementary Table 2). The multiplex immunobead assays were done in 96-well microplate format according to the manufacturer's instructions. A filter-bottom, 96-well microplate (Millipore, Billerica, MA) was blocked for 10 minutes with PBS/bovine serum albumin. Experiments generating standard curves involved a 7-point standard curve using 5-fold serial dilutions from premixed standards provided in the kits. 50 microliters of standards of patient sera was added per well in duplicate and mixed with 50 microliters of the bead mixture. The microplate was incubated for 1 hour at room temperature on a microtiter shaker. Wells were then washed thrice with a washing buffer using a vacuum manifold. Phycoerythrin- (PE-) conjugated secondary antibody was added to the appropriate wells, and the wells were incubated for 45 minutes in the dark with constant shaking. Wells were washed twice, assay buffer was added to each well, and samples were acquired on Luminex xMAP technology (Millipore, St. Charles, MO, USA). Concentrations of each protein were assessed according to standard calibration curves, which analyzed the median fluorescent intensity data with the five-parameter logistic curve fitting method through the MILLIPLEX Analyst Software (Viagene Tech, Carlisle, MA, USA).

### 2.4. Comorbidity, Laboratory, and Clinical Variables

Sociodemographic data (age, sex, and years of education), time on dialysis, medical history, and biochemical data were obtained for all participants by electronic health care system records. The definition of hypertension is 140/90 mmHg or higher or taking antihypertensive drugs. Diabetes is defined as HbA1C 6.5% or higher or taking antidiabetics. History of dyslipidemia and coronary artery disease was based on physician diagnosis. Blood samples were obtained after overnight fasting from patients through the arteriovenous shunt immediately before their scheduled hemodialysis session at a single midweek dialysis session. Biochemical data available for hemodialysis patients included serum values for hemoglobin, albumin, blood urea nitrogen, C-reactive protein, ion calcium, and phosphorous from routine blood samples obtained at the beginning of the hemodialysis session within 30 days before cognitive function assessment.

### 2.5. Control Subjects of the General Population

A voluntary nondialysis comparison group of 37 independently people was recruited by advertisement from the community. Eligibility criteria for the comparison group were the same as for the hemodialysis subjects, with additional exclusions of diagnosed CKD. Sociodemographic data and medical history were obtained for all participants by self-report records. Biochemical data were performed in the same laboratory. Bone turnover biomarkers were measured using Luminex bead-based multiplex assay, and neuropsychological tests (CASI, MoCA, and CES-D) for control subjects were also assessed.

### 2.6. Statistical Analysis

Data were presented as mean ± standard deviation (SD) or percentages. The Kolmogorov-Smirnov test was applied to test for normal distribution. Student's *t*-tests or Mann–Whitney *U* tests were applied to compare means of continuous variables. Categorical data were tested using the chi-squared test. Data were log-transformed to approximate normal distributions. Variance Inflation Factor (VIF) calculation was performed to address the issue of collinearity by using principal component analysis. Screened multiple biomarkers associated with cognitive function were evaluated using linear regression models adjusted for age and sex (equation: cognitive function = *β*0 + *β*1∗log − transformed RANKL + *β*2∗age + *β*3∗sex). We plotted the distribution of log-transformed biomarker values with a neuropsychiatric test score. Correlations between serum bone markers and neuropsychiatric test scores were calculated by Spearman's rank correlation coefficients. The tertile levels of serum bone markers and cognitive function tests (CASI and MoCA) were also analyzed. To identify independent determinants of serum bone markers, multivariate linear regression analysis was performed to assess the relationships among log-transformed biomarkers levels with scores on the MoCA and CASI controlling age, sex, education level, depression scale (CES-D), comorbidities (diabetes mellitus, hypertension, dyslipidemia, and coronary artery disease), clinical laboratory data (hemoglobin, albumin, blood urea nitrogen, ion calcium, phosphorus, parathyroid hormone, alkaline phosphatase, C-reactive protein, and *Kt*/*V*), and hemodialysis duration. Relevant demographic parameters were also analyzed by a backward stepwise selection with *P* values for independent variables to enter and to stay in the models set at 0.2 and, subsequently, a final elimination step at *P* < 0.05. Furthermore, hierarchical selection procedures were also performed to construct a powerful covariate set for adjustment in a sensitivity analysis. Results were reported as a beta coefficient (*β*) with 95% CIs, and a two-tailed *P* < 0.05 was considered statistically significant. All statistical analyses were performed using SAS version 9.4 and STATA version 14.

## 3. Results

### 3.1. Demographic and Clinical Characteristics

**Figure 1:**
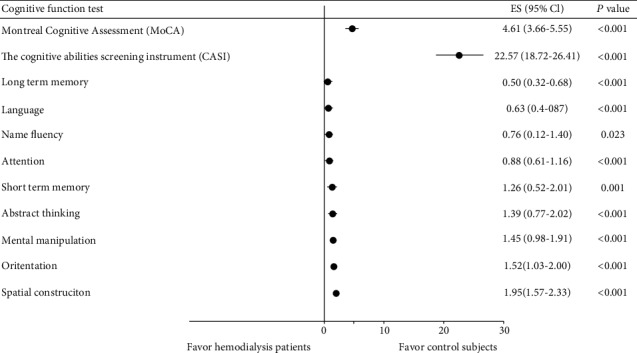
Comparison of cognitive function test (Montreal Cognitive Assessment and Cognitive Abilities Screening Instrument) between the hemodialysis and control subjects in regression analysis.

251 participants in the hemodialysis cohort completed the baseline cognitive assessments included in this analysis. The mean age of the study cohort was 57.8 ± 11.4, 55% were male, 39% had diabetes, 74.5% had hypertension, 34.3% had dyslipidemia, and 21.5% had coronary artery disease (Table 1). The mean years of hemodialysis duration were 6.98 ± 5.89, and most of the vascular access in participants was the arteriovenous fistula (89.6%). The mean serum levels of clinical laboratory data were 10.74 ± 1.22 mg/dl in hemoglobin, 3.90 ± 0.28 mg/dl in albumin, 2.50 ± 4.15 mg/dl in C-reactive protein, 4.62 ± 0.46 mmol/l in ion calcium, 4.67 ± 1.09 mmol/l in phosphate, 66.29 ± 14.09 mg/dl in blood urea nitrogen, and 1.56 ± 0.24 single pool *Kt*/*V* (Table 1). The neuropsychological test scores and serum biomarkers levels based on the Luminex multiplex assay are shown in Table 2. The mean scores of the neuropsychological test were 21.11 ± 5.83 in MoCA and 81.01 ± 16.59 in CASI in hemodialysis participants. All neuropsychological test scores were lower in hemodialysis participants compared to the reference control subjects, except CES-D, CASI long-term memory domain, language domain, and name fluency domain. The mean serum levels of bone turnover biomarkers were 4.62 ± 5.34 ng/ml in RANKL, 1.17 ± 0.42 ng/ml in DKK1, 1.05 ± 1.58 ng/ml in FGF23, 25.60 ± 22.29 ng/ml in Leptin, 14.45 ± 12.93 ng/ml in OC, 7.43 ± 10.66 ng/ml in OPN, 0.98 ± 0.50 ng/ml in OPG, and 3.57 ± 1.40 ng/ml in SOST. All serum biomarker levels were higher in hemodialysis participants compared to the reference control subjects, except DKK1 (Table 2). Comparing the cognitive function test (MoCA and CASI) between hemodialysis patients and control subjects, all cognitive function test scores were lower in hemodialysis patients (Figure 1).

### 3.2. Association of Serum Biomarkers with Cognitive Function in Hemodialysis Participants

**Figure 2:**
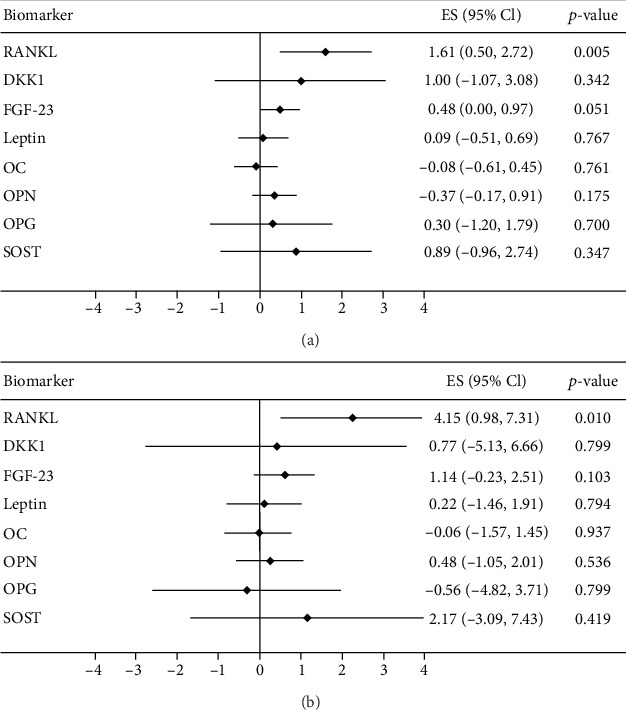
The association of 8 serum bone biomarkers and cognitive function test score based on (a) Montreal Cognitive Assessment and (b) Cognitive Abilities Screening Instrument in participants with hemodialysis. Regression models were adjusted for age and sex.

When relating the bone turnover markers to the neuropsychological test score (MoCA and CASI) one by one in hemodialysis participants adjusting for age and sex, only the RANKL biomarker was significantly and positively related to the cognitive function test score on MoCA and CASI (Figure 2).

### 3.3. Associations of Serum RANKL Levels and Cognitive Impairment in Hemodialysis Participants

**Figure 3:**
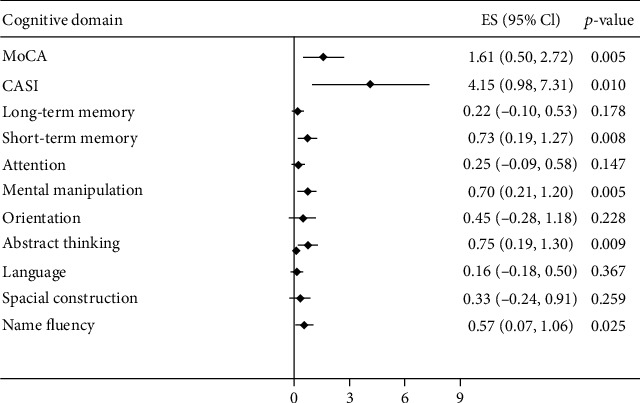
The association between circulating RANKL and cognitive function in patients with hemodialysis. The regression analysis was adjusted for age, sex, and education level.

The scatter plot with cubic spline demonstrated the association of serum RANKL and the cognitive function score on MoCA and CASI scores. There was a positive association between serum log-transformed RANKL levels and cognitive function tests (MoCA and CASI) (Supplementary Figures 2A and 2B). The tertile levels of log-transformed RANKL also demonstrated a positive correlation between higher RANKL levels and better cognitive function tests in CASI and MoCA (Supplementary Figure 3). The positive association was found in different CASI domains, including long-term memory, short-term memory, attention, mental manipulation, abstract thinking, and name fluency (Supplementary Figure 4). However, after adjusting for age, sex, and education levels, the serum RANKL levels were positively correlated with cognitive function test scores on MoCA, CASI, and CASI domains of short-term memory, mental manipulation, abstract thinking, and name fluency (Figure 3). In multiple linear regression models adjusted for age, sex, education levels, depression scales (CES-D), and comorbidities (diabetes mellitus, hypertension, dyslipidemia, and coronary artery disease), higher levels of log-transformed RANKL were associated with better MoCA scores (*β* coefficient 1.12, 95% confidence interval (CI) 0.08 to 2.16; model 2) and better CASI scores (*β* coefficient 2.88, 95% CI 0.27 to 5.49; model 2). Higher serum RANKL levels were found to have an association with better CASI short-term memory domain (*β* coefficient 0.51, 95% CI 0.10 to 0.93; model 2), mental manipulation domain (*β* coefficient 0.51, 95% CI 0.09 to 0.94; model 2), and abstract thinking domain (*β* coefficient 0.56, 95% CI 0.003 to 1.12; model 2) but not name fluency domain (*β* coefficient 0.42, 95% CI -0.06 to 0.89; model 2) (Table 3). Further adjustment for clinical laboratory data (hemoglobin, albumin, ion calcium, phosphorus, parathyroid hormone, alkaline phosphatase, C-reactive protein, and *Kt*/*V*) and hemodialysis duration, serum log-transformed RANKL levels persisted to be positively associated with MoCA scores (*β* coefficient 1.11, 95% CI 0.09 to 2.14; model 3), CASI mental manipulation domain (*β* coefficient 0.53, 95% CI 0.04 to 1.02; model 3), and CASI abstract thinking domain (*β* coefficient 0.59, 95% CI 0.05 to 1.13; model 3). In multiple linear regression models, the cognitive function test score was negatively correlated to age (*β* coefficient -0.19, 95% CI -0.37 to -0.01 in CASI score) and coronary artery disease (*β* coefficient -2.09, 95% CI -3.71 to -0.46 in MoCA score; *β* coefficient -7.08, 95% CI -12.1 to -2.03 in CASI score). Education levels were the strongest correlation factors to the cognitive function test score (*β* coefficient 2.07, 95% CI 1.49 to 2.66 in MoCA score; *β* coefficient 5.55, 95% CI 4.07 to 7.03 in CASI score) (Supplementary Table 3).

We further applied backward stepwise variable selection in the multiple linear analysis, higher levels of log-transformed RANKL were positively correlated to MoCA scores (*β* coefficient 1.14, 95% CI 0.17 to 2.11; model 4), CASI scores (*β* coefficient 3.06, 95% CI 0.24 to 5.88; model 4), CASI short-term memory domain (*β* coefficient 0.52, 95% CI 0.01 to 1.02; model 4), CASI mental manipulation domain (*β* coefficient 0.51, 95% CI 0.05 to 0.96; model 4), and CASI abstract thinking domain (*β* coefficient 0.57, 95% CI 0.06 to 0.96; model 4) (Table 3). Serum RANKL remained independently positively associated with cognitive function in multivariate linear regression analysis with hierarchically covariate selection-adjusted models (Supplementary Table 4).

## 4. Discussion

A multiplex bead-based Luminex assay was used to investigate the association between multiple circulating bone turnover markers and the 2 different cognitive function tests in hemodialysis individuals. The main finding of this study was that serum levels of RANKL were able to classify independently the presence of clinically overt cognitive impairment in hemodialysis patients. Using a linear regression model with age and sex adjustment for multiple biomarker screening, serum RANKL levels were associated with the higher cognitive function, which is defined by MoCA and CASI. In multiple linear regression analysis, serum RANKL levels were positively correlated to the cognitive function test (MoCA and CASI), especially in short-term memory, mental manipulation, and abstract thinking. Other bone turnover makers, including DKK1, FGF23, Leptin, OC, OPN, OPG, and SOST, were statistically insignificantly associated with cognitive function after age and sex adjustment during the screening process. Although one cross-sectional study previously reported that the higher serum FGF23 levels were associated with poor cognitive performance [22], it is unable to determine the associations in our study.

Patients undergoing hemodialysis have more prothrombotic factors, such as endothelial dysfunction, abnormal vascular reactivity, and atherosclerosis, leading to the cause of cognitive impairment. The prevalence of dementia is between 5 and 9% of the population, but there is limited prevalence data in patients with ESKD [23]. The reported prevalence of cognitive impairment among patients with ESKD, as assessed using neuropsychological tests, varies from 16 to 38% [5]. Patients undergoing hemodialysis were found to have more cognitive impairment than the general population, particularly in the domains of orientation and attention and executive function [1]. In the present study, the neuropsychological test scores, including MoCA and CASI, were all lower in hemodialysis participants than in reference control subjects.

Serum RANKL levels were found to be associated with cognitive function test scores in this study. Regarding this finding, RANKL may be considered to have an important role in the pathogenesis of cognitive function in hemodialysis patients. The OPG/RANK/RANKL system is an important pathway in bone biology and skeletal health. This system also plays an important role in other tissues [24, 25], such as the brain, heart, and lungs. The OPG/RANK/RANKL axis has been implicated in the pathophysiology of various cardiovascular disorders that involve a vascular component, such as atherosclerosis and diabetes [26]. However, conflicting results of the OPG/RANK/RANKL axis on cardiovascular disease were reported [27–30]. RANKL was detected in atherosclerotic lesions, potentially promoting inflammation, endothelial cell activation, and matrix degradation [31]. RANKL was reported to exhibit several properties with relevance to atherogenesis, induction of chemotactic properties in monocytes, and induction of matrix metalloproteinase (MMP) activity in vascular smooth muscle cells (SMC) [32, 33]. Raised serum levels of RANKL have been reported in patients with heart failure [31, 34, 35] and predicted the risk of cardiovascular disease in healthy individuals [36]. However, other studies did not find any relation between serum levels of RANKL and cardiovascular disease [27–29]. Besides, an inverse correlation between circulating RANKL and OPG levels was also found [28]. RANKL concentrations also displayed inverse associations with multiple cardiovascular disease risk factors, including smoking, diabetes, and antihypertensive treatment [28]. Furthermore, a significant negative correlation between serum RANKL values and coronary artery calcification score was found in hemodialysis patients [37]. Some basic studies also reported that RANKL was important for dendritic cell survival and, therefore, antigen surveillance, T cell memory formation, and immunomodulatory effects [38]. RANKL also regulates endothelial cell survival and proliferation; disruption along the OPG/RANK/RANKL axis could result in endothelial dysfunction and impaired angiogenesis [38].

RANKL and RANK are also expressed in the central nervous system [39]. In an adult mouse model of ischemic stroke, enhancing RANKL/RANK signaling in wild-type animals by recombinant RANKL is able to significantly reduce the infarct volume [40]. RANKL/RANK is a novel signal involved in the regulation of microglial inflammation through Toll-like receptor (TLR) 4 [40], which is the main damage-associated molecular pattern (DAMP) receptor in the ischemic brain [41]. Both RANKL and RANK are expressed in activated microglia and macrophages (M/M) of ischemic brain tissue, and enhancement of the RANKL/RANK signal using recombinant RANKL (rRANKL) has been shown to reduce ischemic injury in mice [40]. Besides, microglial cells decrease in RANK signaling during inflammation. Pretreatment of the BV2 microglial cell line with rRANKL decreased the ability of Toll-like receptor 3 (TLR3)/Toll-like receptor 4 (TLR4) signaling to activate the expression of TLR3, myeloid differentiation primary response gene 88 (MyD88), and TIR-domain-containing adaptor-inducing interferon- (IFN-) *β* (TRIF). This suppressed BV2 microglial activation, which was confirmed by the reduction of the inflammatory markers iNOS and COX2. Therefore, keeping the expression of adaptor proteins at physiological levels by RANK activation is important to prevent the overactivation of TLR signaling in microglial cells [42]. As we have known, ESKD patients experience more inflammation processes, which may be associated with the decline in neurocognitive performance. RANK signaling plays an important role in the regulation of microglial responses to inflammatory stimuli. This indicated that RANKL could potentially be beneficial in brain damage.

The strength of the present study is that we conducted several detailed neuropsychological assessments. In spite of different sensitivities and specificities of cognitive function tests on MoCA and CASI, similar results using different cognitive function tests may eliminate the bias from the diagnostic tools. We measured multiple bone turnover biomarkers simultaneously using the Luminex bead-based multiplex assay to evaluate the potential biomarkers on cognitive function. However, some limitations still should be addressed. First, our sample came from a single geographic site and patients in this study were receiving hemodialysis; thus, these findings may not be generalizable to patients receiving peritoneal dialysis. Second, brain imaging was not available to ascertain the etiology of cognitive impairment. Thus, we excluded hemodialysis patients with dementia and cerebrovascular disease to decrease potential bias. Third, the concentration of RANKL in cerebrospinal fluid is likely to be more important than serum concentrations with respect to cognitive function; however, this was not assessed in this study. Finally, the cross-sectional study could not infer the causal relationship, so longitudinal studies should be conducted to clarify the issue.

## 5. Conclusion

Serum levels of RANKL were found potentially associated with cognitive function, especially short-term memory, mental manipulation, and abstract thinking in patients receiving hemodialysis. Thus, future studies focusing on pathophysiologic relevance are needed.

## Figures and Tables

**Table 1:** Baseline characteristics of hemodialysis participants.

Patients (*N* = 251)	
Age (years)	57.8 ± 11.4
Male	138 (55.0%)
Education	
No	10 (4.0%)
Elementary school	52 (20.7%)
Junior high school	50 (19.9%)
Senior high school	77 (30.7%)
College	62 (24.7%)
Hemodialysis duration (years)	6.98 ± 5.89
Comorbidities	
Diabetes mellitus	98 (39.0%)
Hypertension	187 (74.5%)
Dyslipidemia	86 (34.3%)
Coronary artery disease	54 (21.5%)
Clinical laboratory data	
Hemoglobin (mg/dl)	10.74 ± 1.22
Albumin (mg/dl)	3.9 ± 0.28
C-reactive protein (mg/dl)	2.5 ± 4.15
Ion calcium (mmol/l)	4.62 ± 0.46
Phosphate (mmol/l)	4.67 ± 1.09
Blood urea nitrogen (mg/dl)	66.29 ± 14.09
Single pool *Kt*/*V*	1.56 ± 0.24

^∗^Other causes of end-stage renal disease include polycystic kidney disease, tumor, IgA nephropathy, systemic lupus erythematosus, gout, and interstitial nephritis.

**Table 2:** Neuropsychiatric test and multiplex bead-based immunoassay of bone turnover markers in hemodialysis participants and reference control subjects.

	Hemodialysis participants (*N* = 251)	Reference control subjects (*N* = 37)	*P*
Neuropsychiatric test			
Montreal Cognitive Assessment (MoCA)	21 ± 6	25 ± 3	<0.001
Cognitive Abilities Screening Instrument (CASI)	81 ± 17	90 ± 6	<0.001
(i) Long-term memory	9 ± 2	10 ± 0	0.051
(ii) Short-term memory	8 ± 3	9 ± 3	0.018
(iii) Attention	7 ± 2	8 ± 1	0.006
(iv) Mental manipulation	8 ± 3	9 ± 1	<0.001
(v) Orientation	16 ± 4	18 ± 1	0.037
(vi) Abstract thinking	8 ± 3	9 ± 2	0.022
(vii) Language	9 ± 2	10 ± 1	0.06
(viii) Spatial construction	8 ± 3	10 ± 1	<0.001
(ix) Name fluency	7 ± 3	8 ± 2	0.07
Center for Epidemiological Studies Depression Scale (CES-D)	11 ± 7	12 ± 7	0.54
Bone turnover markers			
Receptor activator of nuclear factor kappa-Β ligand (RANKL) (ng/ml)	4.62 ± 5.34	2.65 ± 1.20	0.029
Dickkopf-related protein 1 (DKK1) (ng/ml)	1.17 ± 0.42	1.18 ± 0.31	0.99
Fibroblast growth factor 23 (FGF23) (ng/ml)	1.05 ± 1.58	0.01 ± 0.003	<0.001
Leptin (ng/ml)	25.60 ± 22.29	9.86 ± 15.67	<0.001
Osteocalcin (OC) (ng/ml)	14.45 ± 12.93	2.56 ± 8.7	<0.001
Osteopontin (OPN) (ng/ml)	7.43 ± 10.66	2.89 ± 2.69	0.012
Osteoprotegerin (OPG) (ng/ml)	0.98 ± 0.50	0.28 ± 0.08	<0.001
Sclerostin (SOST) (ng/ml)	3.57 ± 1.40	1.97 ± 0.68	<0.001

**Table 3:** Association between log-transformed serum RANKL concentrations and cognitive function test in hemodialysis participants using linear regression analysis with confounder adjustment.

Cognitive test	Linear regression models			
	Model 1	Model 2	Model 3	Model 4
MoCA	1.19 (0.11 to 2.27)	1.12 (0.08 to 2.16)	1.11 (0.09 to 2.14)	1.14 (0.17 to 2.11)
CASI	2.98 (0.13 to 5.82)	2.88 (0.27 to 5.49)	2.83 (-0.12 to 5.78)	3.06 (0.24 to 5.88)
CASI short-term memory	0.55 (0.11 to 0.99)	0.51 (0.10 to 0.93)	0.53 (-0.001 to 1.06)	0.52 (0.01 to 1.02)
CASI mental manipulation	0.56 (0.11 to 1.00)	0.51 (0.09 to 0.94)	0.53 (0.04 to 1.02)	0.51 (0.05 to 0.96)
CASI abstract thinking	0.56 (-0.02 to 1.14)	0.56 (0.003 to 1.12)	0.59 (0.05 to 1.13)	0.57 (0.06 to 1.09)
CASI name fluency	0.43 (-0.04 to 0.90)	0.42 (-0.06 to 0.89)	0.28 (-0.21 to 0.78)	0.29 (-0.19 to 0.76)

Note: CASI: Cognitive Abilities Screening Instrument; MoCA: Montreal Cognitive Assessment. The multivariable linear model demonstrated as a beta coefficient (*β*) with 95% confidence intervals (CIs). Model 1 is adjusted for age, sex, and education level. Model 2 is adjusted for age, sex, education level, depression scale, and comorbidity (diabetes mellitus, hypertension, dyslipidemia, and coronary artery disease). Model 3 is adjusted for age, sex, education level, depression scale, comorbidity (diabetes mellitus, hypertension, dyslipidemia, and coronary artery disease), clinical laboratory data (hemoglobin, albumin, ion calcium, phosphorous, C-reactive protein, parathyroid hormone, alkaline phosphatase, and *Kt*/*V*), and hemodialysis duration. Model 4 is adjusted for stepwise procedure selected covariates. Covariate selection of age, education, depression scale, coronary artery disease, blood urea nitrogen, ion calcium, phosphate, and C-reactive protein in MoCA; covariate selection of age, sex, education, diabetes, coronary artery disease, blood urea nitrogen, albumin, ion calcium, and C-reactive protein in CASI; covariate selection of age, sex, education, depression scale, diabetes, coronary artery disease, albumin, ion calcium, phosphate, and parathyroid hormone in CASI short-term memory; covariate selection of education, depression scale, hyperlipidemia, and iron calcium in CASI mental manipulation; covariate selection of age, sex, education, depression scale, diabetes, coronary artery disease, albumin, ion calcium, phosphate, and parathyroid hormone in CASI short-term memory; covariate selection of sex, education, diabetes, coronary artery disease, blood urea nitrogen, and C-reactive protein in CASI abstract thinking; covariate selection of age, education, diabetes, coronary artery disease, albumin, and alkaline phosphatase in CASI name fluency.

## Data Availability

The data used to support the findings of this study are available from the corresponding author upon request.

## Abbreviations

RANKL: Receptor activator of nuclear factor kappa-B ligand
CKD: Chronic kidney disease
CASI: Cognitive Abilities Screening Instrument
MoCA: Montreal Cognitive Assessment.

## Supplementary Materials

Supplementary Materials The MILLIPLEX MAP technology (Luminex) method. Supplementary Table 1: description of the neuropsychiatric test on patients with hemodialysis in the study. Supplementary Table 2: list of bone turnover biomarkers measured by bead system multiplex Luminex immunoassays. Supplementary Table 3: multiple linear regression analysis of cognitive function test (Montreal Cognitive Assessment and Cognitive Abilities Screening Instrument). Supplementary Table 4: association between log-transformed serum RANKL concentrations and cognitive function test in hemodialysis participants using linear regression analysis adjusted for hierarchically selected covariates. Supplementary Figure 1: study flowchart. Supplementary Figure 2: scatter plot with cubic spline demonstrated the association of log-transformed circulating receptor activator of nuclear factor kappa-B ligand (RANKL) levels and cognitive function test (MoCA and CASI). Supplementary Figure 3: the tertile levels of the log-transformed RANKL and cognitive function tests of CASI and MoCA. Supplementary Figure 4: the tertile levels of log-transformed RANKL and different domains of the CASI test.
