# Supplementary material for: Associations of Bone Turnover Markers with Cognitive Function in Patients Undergoing Hemodialysis
Source: Dis Markers. 2020 Apr 24;2020:8641749. doi: 10.1155/2020/8641749 (PMC7196142; doi:10.1155/2020/8641749)

**Supplementary material**

**Supplementary Table 1.** Description of the neuropsychiatric test on patients with hemodialysis in the study

| Neuropsychiatric test | Description |
| --- | --- |
| Montreal Cognitive Assessment (MoCA) | The MoCA is a brief screening instrument assessing several cognitive domains including attention, executive functions, language, memory, and orientation. It is available in several languages and is widely used to detect cognitive impairment in numerous neurological and non-neurological diseases. |
| Cognitive Abilities Screening Instrument (CASI) | The CASI is a 40-item global cognitive test that assesses a broad range of cognitive domains. It has nine cognitive evaluation domain including long-term memory, short term memory, orientation, attention, mental manipulation, list-generating fluency, language, abstraction/judgment, and drawing. |
| Center for Epidemiologic Studies Depression Scale (CES-D) | This scale is used to assess the frequency of depression symptoms over the past week. Points assigned for response options were 0 for “rarely or none of the time,” 1 for “some or a little of the time,” 2 for “occasionally or a moderate amount of time” and 3 for “most or all of the time,” with positively phrased items reverse coded. |

**Supplementary Table 2.** List of bone turnover biomarkers measured by beads system multiplex Luminex immunoassays.

| MILLIPLEX^®^ map Kits | Catalog No. | Protein Analyte | UniProt ID |
| --- | --- | --- | --- |
| Human RANKL Single Plex | HRNKLMAG-51K-01 | Receptor activator of nuclear factor kappa-Β ligand (RANKL) | O14788 |
| Human Bone Panel | HBNMAG-51K | Dickkopf-related protein 1 (DKK1) | O94907 |
| Human Bone Panel | HBNMAG-51K | Fibroblast growth factor 23 (FGF23) | Q9GZV9 |
| Human Bone Panel | HBNMAG-51K | Leptin | O15243 |
| Human Bone Panel | HBNMAG-51K | Osteocalcin (OC) | P02818 |
| Human Bone Panel | HBNMAG-51K | Osteopontin (OPN) | P10451 |
| Human Bone Panel | HBNMAG-51K | Osteoprotegerin (OPG) | O00300 |
| Human Bone Panel | HBNMAG-51K | Sclerostin (SOST) | Q9BQB4 |

**Supplementary Table 3.** Multiple linear regression analysis of cognitive function test (Montreal Cognitive Assessment and The Cognitive Abilities Screening Instrument)

|  | MoCA | | | CASI | | |
| --- | --- | --- | --- | --- | --- | --- |
| Dependent variable | β coefficient | 95% CI | P value | β coefficient | 95% CI | P value |
| log RANKL | 1.11 | 0.09 to 2.14 | 0.034 | 2.83 | -0.12 to 5.78 | 0.060 |
| Age | -0.05 | -0.11 to 0.01 | 0.13 | -0.19 | -0.37 to -0.02 | 0.033 |
| Sex | -0.38 | -1.71 to 0.95 | 0.58 | -2.13 | -5.95 to 1.69 | 0.270 |
| Education | 2.00 | 1.36 to 2.64 | <0.001 | 5.31 | 3.47 to 7.14 | <0.001 |
| CESD score | -0.08 | -0.16 to 0.01 | 0.093 | -0.12 | -0.37 to 0.12 | 0.330 |
| Diabetes | -0.90 | -2.28 to 0.47 | 0.20 | -4.21 | -8.15 to -0.27 | 0.036 |
| Hypertension | 0.83 | -0.73 to 2.40 | 0.30 | -0.19 | -4.70 to 4.32 | 0.930 |
| Coronary artery disease | -2.07 | -3.66 to -0.48 | 0.011 | -7.01 | -11.6 to -2.44 | 0.003 |
| Hyperlipidemia | -0.04 | -1.43 to 1.35 | 0.95 | -2.07 | -6.08 to 1.93 | 0.310 |
| Hemoglobin | -0.08 | -0.62 to 0.46 | 0.77 | -0.73 | -2.28 to 0.82 | 0.350 |
| Albumin | 1.09 | -1.40 to 3.57 | 0.39 | 4.80 | -2.30 to 11.9 | 0.180 |
| Ion Calcium | -1.49 | -2.95 to -0.02 | 0.046 | -3.53 | -7.75 to 0.69 | 0.100 |
| Phosphate | 0.39 | -0.20 to 0.98 | 0.20 | 0.14 | -1.54 to 1.83 | 0.870 |
| Hemodialysis duration | 0.03 | -0.08 to 0.14 | 0.62 | -0.07 | -0.39 to 0.25 | 0.680 |
| C-reactive protein | 0.09 | -0.08 to 0.26 | 0.28 | 0.21 | -0.26 to 0.67 | 0.380 |
| Blood urea nitrogen | 0.05 | 0.00 - 0.10 | 0.038 | 0.09 | -0.04 - 0.23 | 0.180 |
| Parathyroid hormone | 0.0006 | -0.001 - 0.004 | 0.50 | 0.002 | -0.003 - 0.007 | 0.450 |
| Alkaline phosphatase | -0.001 | -0.005 - 0.004 | 0.54 | -0.005 | -0.03 - 0.007 | 0.420 |
| Kt/V | -0.24 | -2.98 - 2.50 | 0.86 | 1.84 | -6.04 - 9.72 | 0.650 |

Receptor activator of nuclear factor kappa-Β ligand, RANKL; Center for Epidemiological Studies Depression, CESD

**Supplementary Table 4.** Association between log-transformed serum RANKL concentrations and cognitive function test in hemodialysis participants using linear regression analysis adjusted for hierarchically selected covariates

| Cognitive test | Linear regression models |
| --- | --- |
| MoCA | 1.63 (0.51 to 2.76) |
| CASI | 4.28 (1.08 to 7.48) |
| CASI - Short term Memory | 0.53 (0.02 to 1.04) |
| CASI - Mental manipulation | 0.52 (0.06 to 0.98) |
| CASI - Abstract thinking | 0.57 (0.05 to 1.09) |
| CASI - Name fluency | 0.58 (0.08 to 1.07) |

Note: CASI, Cognitive Abilities Screening Instrument; MoCA, Montreal Cognitive Assessment

The multivariable linear model demonstrated as a Beta coefficient (β) with 95% Confidence intervals (CIs)

Model is adjusted hierarchically selected covariates

Covariates selection of age in MoCA; Covariates selection of age in CASI; Covariates selection of age, sex, education, depression scale, and diabetes in CASI - Short term Memory; Covariates selection of age, sex, education, and depression scale in CASI - Mental manipulation; Covariates selection of age, sex, and education in CASI - Abstract thinking; Covariates selection of age in CASI - Name fluency

**Supplementary Figure 1.** Study flowchart


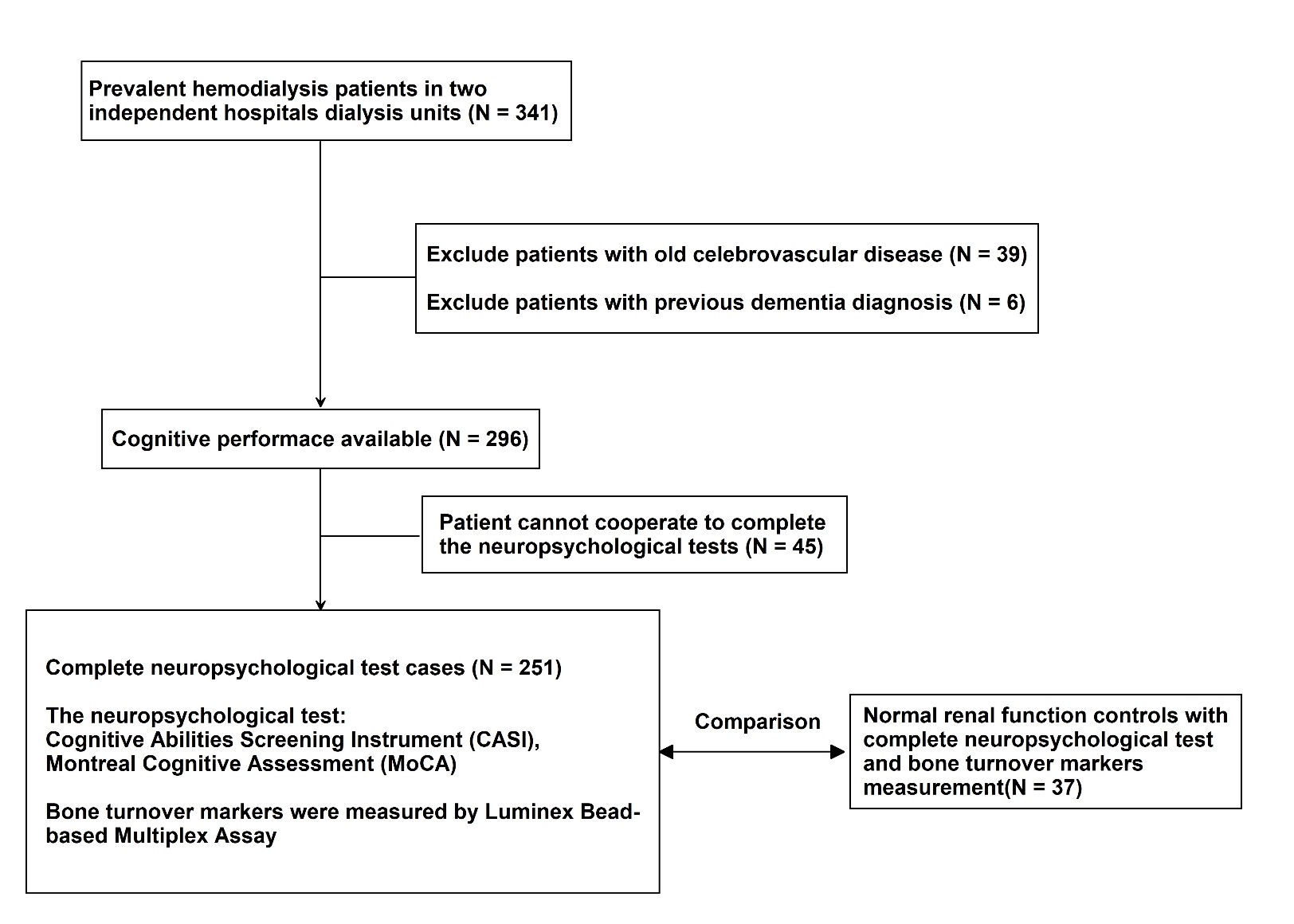


**Supplementary Figure 2.** Scatter plot with cubic spline demonstrated the association of log-transformed circulating Receptor activator of nuclear factor kappa-B ligand (RANKL) levels and cognitive function test (MoCA and CASI)


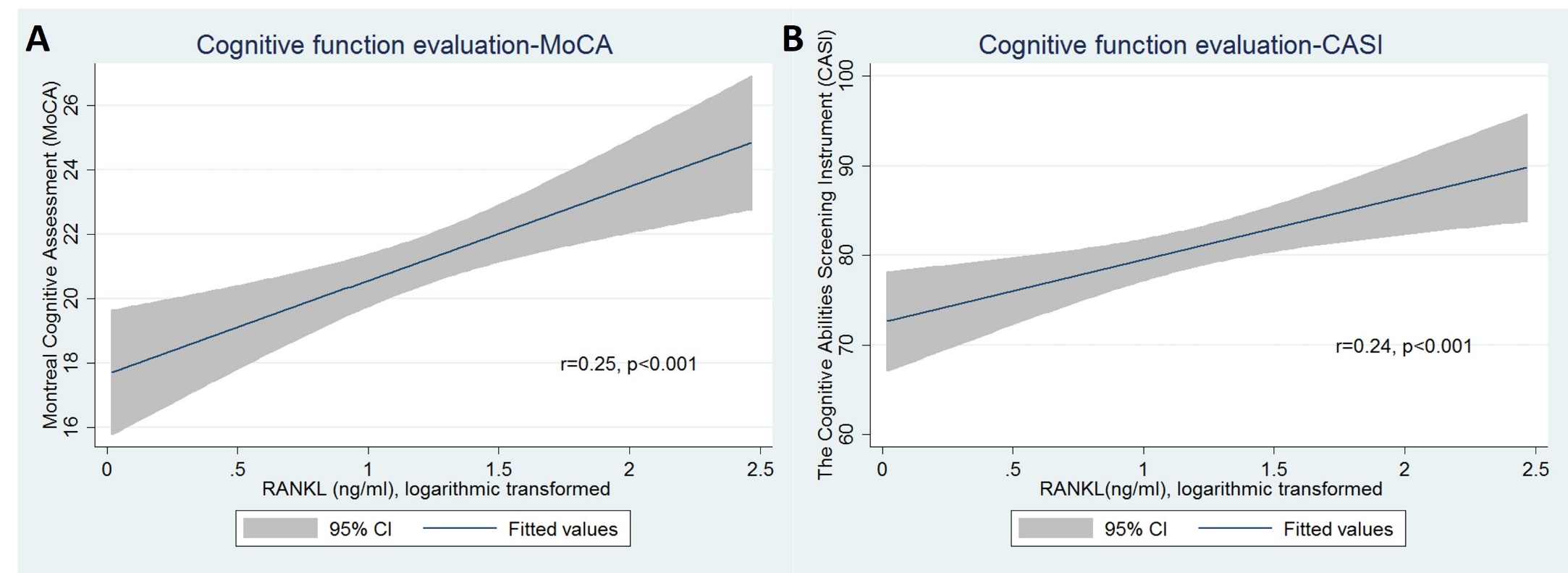


**Supplementary Figure 3.** The tertile levels of log-transformed RANKL and cognitive function test of CASI and MoCA


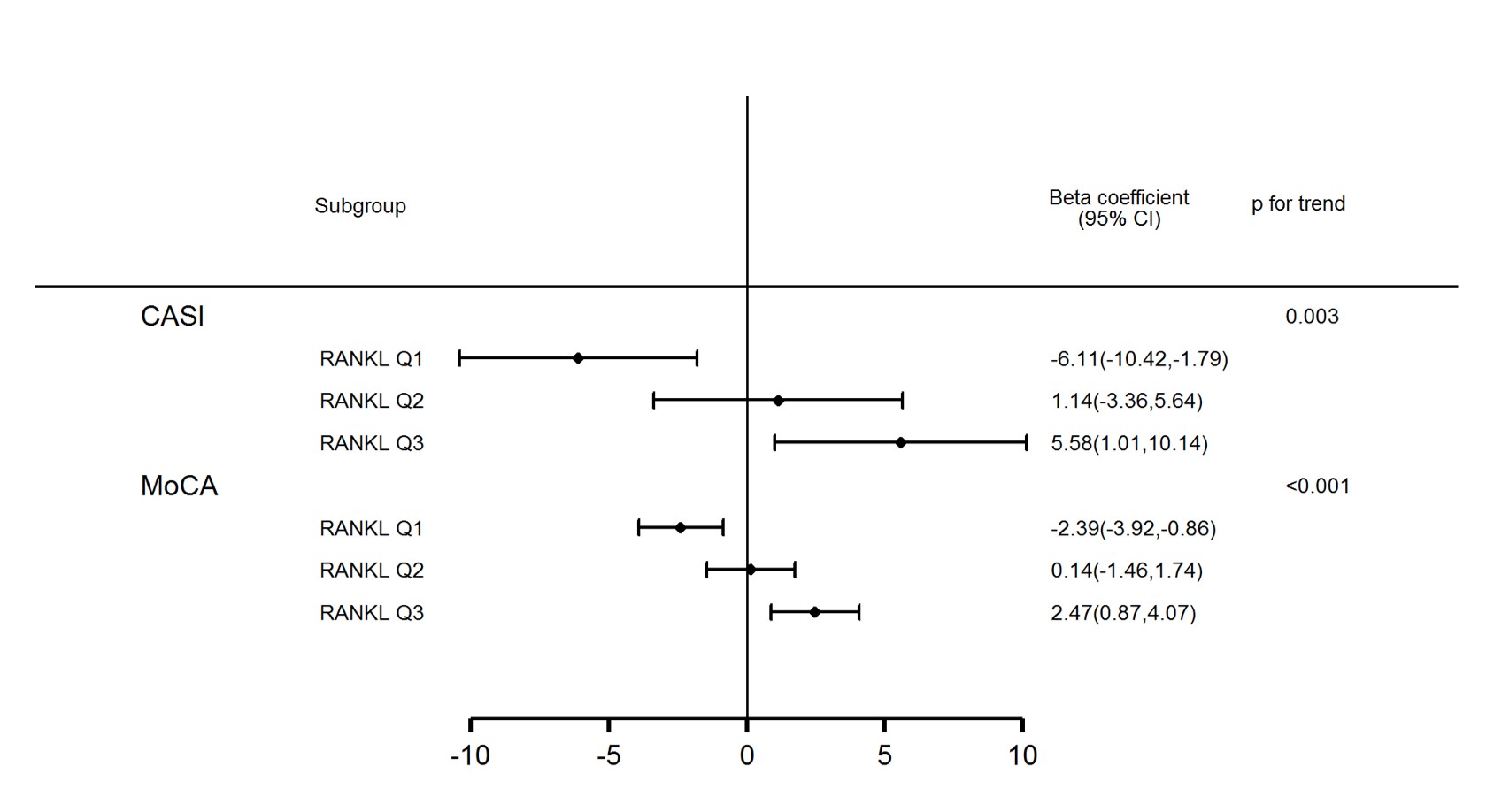


**Supplementary Figure 4.** The tertile levels of log-transformed RANKL and different domain of CASI test
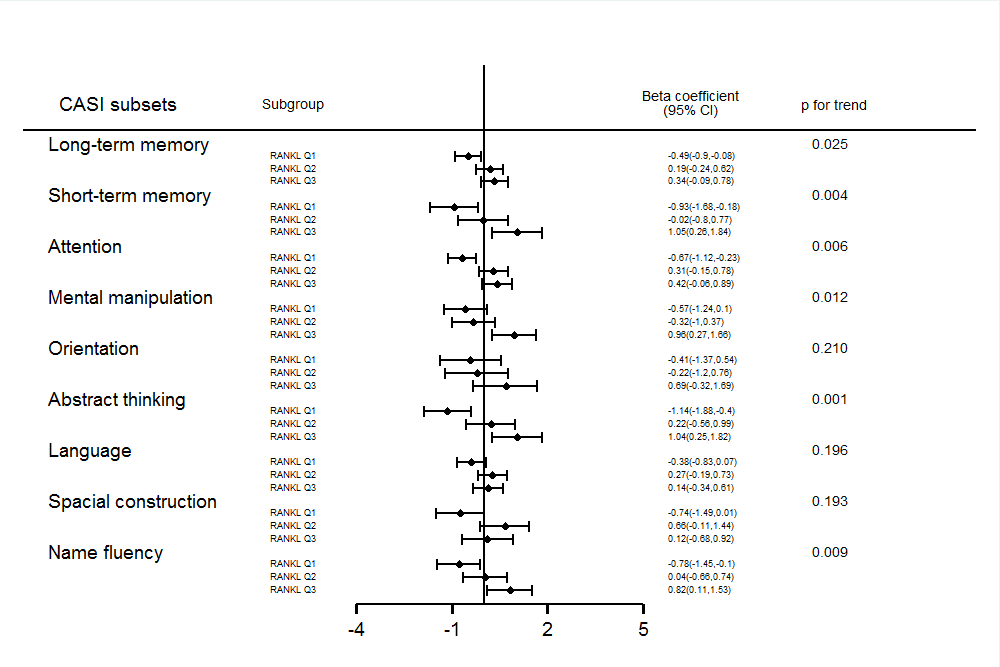

Supplement: Supplementary Materials — The MILLIPLEX MAP technology (Luminex) method. Supplementary Table 1: description of the neuropsychiatric test on patients with hemodialysis in the study. Supplementary Table 2: list of bone turnover biomarkers measured by bead system multiplex Luminex immunoassays. Supplementary Table 3: multiple linear regression analysis of cognitive function test (Montreal Cognitive Assessment and Cognitive Abilities Screening Instrument). Supplementary Table 4: association between log-transformed serum RANKL concentrations and cognitive function test in hemodialysis participants using linear regression analysis adjusted for hierarchically selected covariates. Supplementary Figure 1: study flowchart. Supplementary Figure 2: scatter plot with cubic spline demonstrated the association of log-transformed circulating receptor activator of nuclear factor kappa-B ligand (RANKL) levels and cognitive function test (MoCA and CASI). Supplementary Figure 3: the tertile levels of the log-transformed RANKL and cognitive function tests of CASI and MoCA. Supplementary Figure 4: the tertile levels of log-transformed RANKL and different domains of the CASI test. [file 8641749.f1.docx]
